# Ambulatory-Based Standardized Therapy for Multi-Drug Resistant Tuberculosis: Experience from Nepal, 2005–2006

**DOI:** 10.1371/journal.pone.0008313

**Published:** 2009-12-23

**Authors:** Pushpa Malla, Elisabeth Eva Kanitz, Mohammad Akhtar, Dennis Falzon, Knut Feldmann, Christian Gunneberg, Shyam Sundar Jha, Bhagwan Maharjan, Mohan Kumar Prasai, Bhabana Shrestha, Sharat Chandra Verma, Matteo Zignol

**Affiliations:** 1 National Tuberculosis Centre (NTC), Ministry of Health and Population, Kathmandu, Nepal; 2 World Health Organization, Geneva, Switzerland; 3 World Health Organization, Country Office, Kathmandu, Nepal; 4 Kuratorium Tuberkulose in der Welt, Gauting, Germany; 5 German Nepal Tuberculosis Project (GENTUP), Kathmandu, Nepal; 6 Regional Tuberculosis Center, Pokhara, Nepal; McGill University, Canada

## Abstract

**Objective:**

The aim of this study was to describe treatment outcomes for multi-drug resistant tuberculosis (MDR-TB) outpatients on a standardized regimen in Nepal.

**Methodology:**

Data on pulmonary MDR-TB patients enrolled for treatment in the Green Light Committee-approved National Programme between 15 September 2005 and 15 September 2006 were studied. Standardized regimen was used (8Z-Km-Ofx-Eto-Cs/16Z-Ofx-Eto-Cs) for a maximum of 32 months and follow-up was by smear and culture. Drug susceptibility testing (DST) results were not used to modify the treatment regimen. MDR-TB therapy was delivered in outpatient facilities for the whole course of treatment. Multivariable analysis was used to explain bacteriological cure as a function of sex, age, initial body weight, history of previous treatment and the region of report.

**Principal Findings:**

In the first 12-months, 175 laboratory-confirmed MDR-TB cases (62% males) had outcomes reported. Most cases had failed a Category 2 first-line regimen (87%) or a Category 1 regimen (6%), 2% were previously untreated contacts of MDR-TB cases and 5% were unspecified. Cure was reported among 70% of patients (range 38%–93% by Region), 8% died, 5% failed treatment, and 17% defaulted. Unfavorable outcomes were not correlated to the number of resistant drugs at baseline DST. Cases who died had a lower mean body weight than those surviving (40.3 kg vs 47.2 kg, p<0.05). Default was significantly higher in two regions [Eastern OR = 6.2; 95%CL2.0-18.9; Far West OR = 5.0; 95%CL1.0-24.3]. At logistic regression, cure was inversely associated with body weight <36 kg [Adj.OR = 0.1; 95%CL0.0-0.3; ref. 55–75 kg] and treatment in the Eastern region [Adj.OR = 0.1; 95%CL0.0-0.4; ref. Central region].

**Conclusions:**

The implementation of an ambulatory-based treatment programme for MDR-TB based on a fully standardized regimen can yield high cure rates even in resource-limited settings. The determinants of unfavorable outcome should be investigated thoroughly to maximize likelihood of successful treatment.

## Introduction

The emergence of strains of tuberculosis (TB) that resist drugs poses a potentially devastating threat to TB control globally [1], [2]. Forms of TB resistant to the most effective anti-TB medication, including multidrug-resistant TB (MDR-TB, defined as TB resistant to at least isoniazid and rifampicin, the two most powerful anti-TB drugs) [3] and extensively drug-resistant TB (XDR-TB, defined as MDR-TB plus resistance to at least fluoroquinolones and one of the second-line injectable drugs - amikacin, kanamycin or capreomycin) [4], have been identified in many parts of the world. Drug-resistant TB is associated with inadequate treatment, resulting from too few or low-quality drugs, non-compliance on part of the patient, and conditions which favour TB transmission. These factors are particularly common in resource-limited settings.

Treatment of MDR-TB is resource intensive and lasts for 24 months or more, requiring a combination of second-line drugs that are more expensive, less effective and more toxic than those used in standard first-line treatment regimens [3], [5]. The control of drug-resistant TB requires a strong health infrastructure to ensure prompt diagnosis, timely delivery of effective treatment, and interventions to reduce transmission, while monitoring the development of the epidemic through surveillance activities [3]. In 2000, to address the emergence of MDR-TB, the World Health Organization (WHO) and the STOP TB Partnership formed a subgroup called the Green Light Committee (GLC) Initiative whose mission is to ensure effective treatment of patients with drug-resistant TB in resource-limited settings in accordance with WHO guidelines [6]. Since its establishment the GLC has approved second-line treatment for over 50,000 MDR-TB patients in more than 60 countries [7]. One of these countries is Nepal, where a GLC-approved programme started treating patients in 2005.

**Figure 1:**
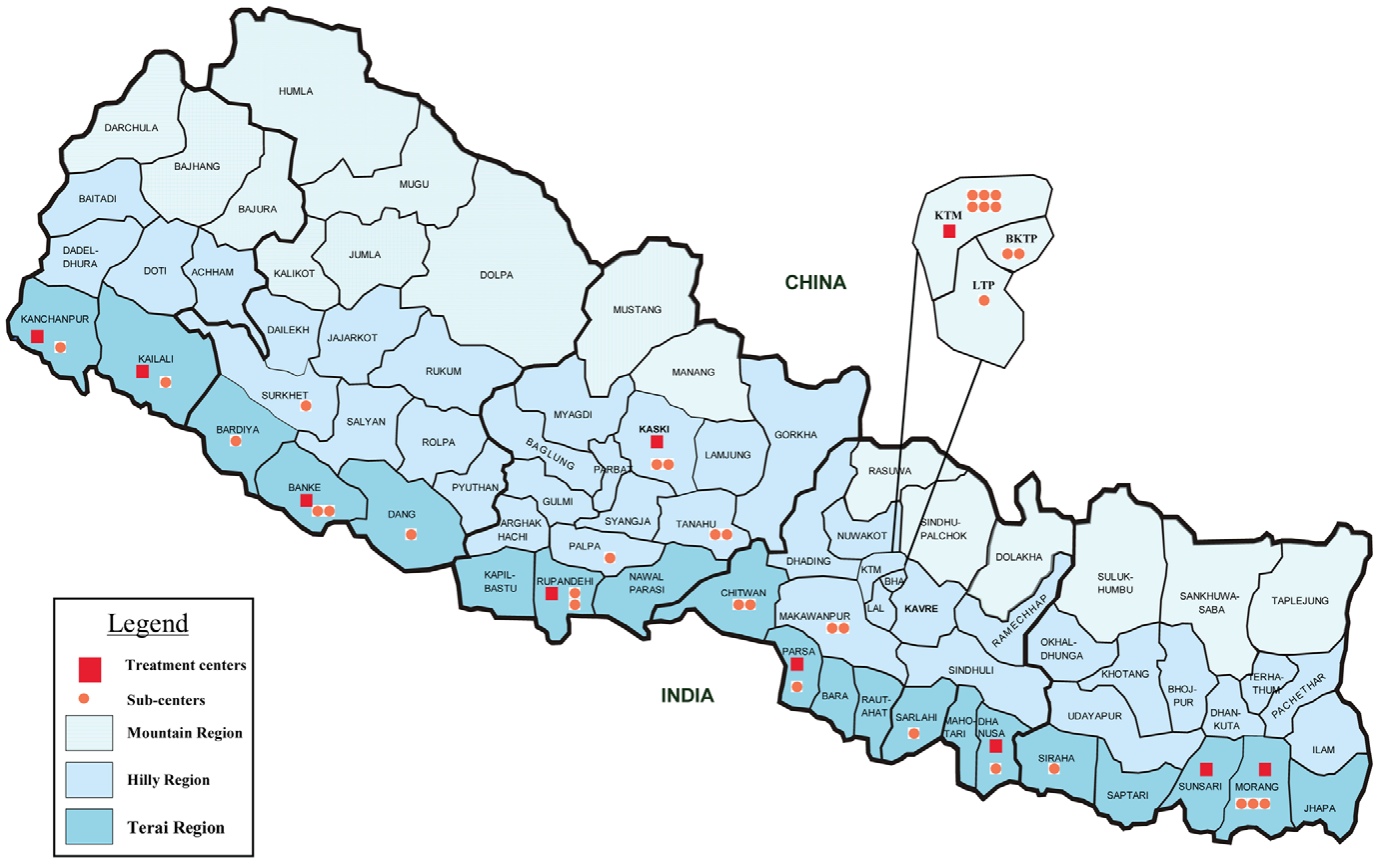
MDR-TB treatment centers and sub-centers in Nepal. Footnote Figure 1: In 2005–2006, not all the health centres were functioning. The 175 cases included in this study were under care at 17 clinics (Bhaktapur, Bheri, Bir, Genetup, Haraicha, HelpHand, INF_Banke, KohalpurMC, Mahakali, Mangalbare, NATA, NMC, NTC, Patan, RTC, Stupa, and TUTH).

In Nepal TB is a major public health issue. In 2007, there were an estimated 48,766 incident TB cases (173/100,000) [8]. MDR-TB occurred among 2.9% of previously un-treated TB cases and 11.7% of previously treated [1], [9]. In 2001–2, 88% of cases in whom a retreatment regimen (“Category 2”) failed as well as 24% in whom regimen for previously untreated (“Category 1”) failed were found to have MDR-TB [9]. To address this challenge, National TB Programme designed a standardized retreatment regimen for MDR-TB with second-line drugs based on surveyed drug resistance patterns in the country [10]. Patients were eligible for such regimen for MDR-TB if they failed a retreatment regimen with first line drugs (ahead of laboratory confirmation) or if they were confirmed MDR-TB, while Category 1 failures and smear positive contacts of MDR-TB patients would be tested for DST before start of MDR-TB treatment. Drugs to counter and prevent side-effects were made available free-of-charge to patients. NTP staff were trained to manage the patients and record data for the programme. Treatment was delivered under direct observation on an ambulatory basis through a decentralized network of clinics. By June 2009, 612 patients had been started on treatment and 10 treatment centres and 34 sub-centers were in use throughout the country (Figure 1). The Central region - which includes the capital Kathmandu - accounts for more than half of the patients enrolled and has been the longest standing DOTS-Plus centre.

In this article we describe the outcomes of patients with laboratory confirmed MDR-TB who received treatment during the first 12-months of the MDR-TB treatment programme.

## Materials and Methods

### Study Population

Between 15 September 2005 and 15 September 2006 a total of 224 patients were enrolled in the GLC-approved treatment programme in Nepal to receive standardized, second-line drug therapy for pulmonary MDR-TB. Of these, 175 patients with confirmed MDR-TB who began treatment in this period were retained for the study (Table 1). Exclusions included eligible patients who did not start treatment (2 cases), patients with no culture results reported after treatment initiation (2 cases), and those with negative culture results at the beginning and throughout the treatment period (18 cases). Of the 202 patients with culture positive TB, 8 were found to have Nontuberculous mycobacteria (NTM). Of the 194 patients with disease caused by *Mycobacterium tuberculosis* we excluded those with no baseline drug susceptibility testing (DST) results available (6 cases), and those whose baseline DST results did not meet the definition of MDR-TB (confirmed susceptibility to either isoniazid and rifampicin) (13 cases).

**Table 1:** Selection of the study population.

**Enrolled between 15 September 2005 and 15 September 2006**	→	224 patients	→	2 patients never started on treatment
		↓		
**Treated**	→	222 patients	→	18 culture negative
		↓	→	2 culture contaminated
				
**With culture positive results**	→	202 patients	→	5 *M.intracellulare*
			→	2 *M.fortuitum*
		↓	→	1 *M.avium*
				
**Confirmed M.tb**	→	194 patients	→	6 cases with DST on specimens taken >60 days before or >40 days after start of treatment
			→	5 rifampicin sensitive and isoniazid resistant
		↓	→	8 rifampicin and isoniazid sensitive
**Confirmed MDR-TB**	→	175 patients		

Patients were categorized according to the registration groups used by the Nepal NTP: Category 2 treatment failure, Category 1 treatment failure with DST confirmed MDR-TB, and previously untreated MDR-TB family contact. Cases for whom the registration group was not known were retained in the analysis.

This analysis was carried out retrospectively and involved basic data collected on a routine basis within the NTP services to regularly monitor the performance of the MDR-TB treatment program. The data were analyzed anonymously. The implementation of such program activity, including the monitoring and evaluation system, is a routine and regular component of the NTP activities and approved by the Ministry of Health and Population of Nepal; therefore no ethical approval was required for this analysis.

### Laboratory Diagnosis and Bacteriological Follow-Up

Diagnosis of MDR-TB was performed at GENETUP TB-laboratory which functions as the National Reference Laboratory in the capital Kathmandu using the indirect proportion method on Löwenstein-Jensen medium. The external quality control for this laboratory is organized and performed by the Supranational Laboratory in Gauting, Germany since 1994 using proportion method. At least one positive culture per patient was also tested using the GenoType® MTBDR plus test of Hain Lifescience. All discrepant results were retested in both systems. If the identification of strains in the GenoType® MTBDR plus test revealed not a *M. tuberculosis complex*, the GenoType® Mycobacteria CM test was applied additionally to identify the NTM. In the study period DST was available for the following four first-line drugs: rifampicin, isoniazid, streptomycin, and ethambutol. DST for pyrazinamide and second-line drugs was not available in the country at the time of this study. In this article, DST was considered as baseline if the sample (sputum) was obtained from a patient within a period of 60 days prior to starting treatment until 40 days after start.

Laboratory follow-up to monitor treatment progress is conducted by sputum smear microscopy and culture conducted monthly during the first eight months of treatment (or 12 months if intensive phase was extended) and then every two months till the end of treatment.

### Treatment Scheme and Delivery

All patients enrolled received the same standardized treatment regimen for MDR-TB. Individual DST results were not used to modify the treatment regimen. The regimen was designed based on the results of DST to first- and second-line anti-TB drugs conducted within the context of a nationwide drug resistance survey, as described elsewhere [9], [10], and in accordance with experts' opinion from the GLC. In addition the database of GENETUP TB-laboratory was reviewed to inform the decision making process. The regimen is composed of five drugs (pyrazinamide, kanamycin, ofloxacin, ethionamide, and cycloserine) in the intensive phase, which usually lasts eight months but is extended to twelve months if the patient is smear- or culture-positive at six months (8Z-Km-Ofx-Eto-Cs/16Z-Ofx-Eto-Cs). It should be noted that drugs were selected in accordance to the 2003 WHO guidelines for TB treatment [11]. At that time ofloxacin was the recommended fluoroquinolone. Cycloserine was selected instead of PAS because felt to cause less serious adverse events and consequently be more manageable in a fully ambulatory-based treatment program. In addition, at that time, PAS was only available through the GLC mechanism in format that required refrigeration and this was felt a serious barrier for its use in Nepal.

The duration of the initial phase was also discussed with the GLC. In Nepal is was decided to prolong the initial phase to 8 months to allow the administration of kanamycin over a longer period. It was felt that to provide the full dosage of daily 6 months kanamycin over a slightly longer period (4 month daily and 4 months thrice weekly) - 8 months in total, might reduce the toxic effects that are usually seen between 4 to 6 months, and extend the time of impact on the overall regimen.

The same drugs, excepting kanamycin, are used in the continuation phase, which usually lasts 16 months but may be extended by up to 8 months if the patient's culture converts to negative only between 12 and 18 months of treatment. Treatment is available in two dosages according to the weight of the patient at the beginning of treatment (up to 50 kg, >50 kg). The weight categories were carefully worked out using the spread of actual weights of a large sample of previously treated TB patients, and showed to work well in practice. Weight cut-off were similar to those used in another study in the same geographical area [12].

Treatment is delivered daily at the clinic. Support during treatment is guaranteed by the presence of a treatment support person nominated by the patient. A gastroprotective agent (ranitidine) is also taken daily during the course of treatment to decrease side effects. Treatment of minor adverse reactions with symptomatic drugs as well as admission to hospital for severe side effects are free of costs for the patients.

An active defaulter tracing system is in place and implemented locally by the clinics with support and supervision from the District TB/Leprosy Officers.

### Data Recording and Analysis

Patient data are kept peripherally on treatment cards, key outcome and treatment information transferred to district MDR-TB registers and from these reported in aggregated quarterly reports. Data from all the district registers is transferred to the national MDR-TB treatment register, which is kept and updated at the National Tuberculosis Centre (NTC) in Kathmandu. This includes demographic information, initial body weight, patient category based on previous treatment, treatment starting date, bacteriology and outcome (dated). Information on socioeconomic background, co-morbidities, or compliance with treatment was not available from this source. An electronic register based on Open Medical Record System (openmrs.org/wiki/Mdrtb_Module) was installed for use at the NTC in October-November 2008 and updated retrospectively with data from patients started in the national MDR-TB treatment programme since 2005.

After cleaning the data were analyzed using the Stata SE/10.0 (Texas, US, 2007) and the Statcalc function of EpiInfo version 6 (CDC). Chi-squared test was used to identify significant associations (p<0.05) between categorical variables and outcomes and to test linear trend. Strength of association is shown as crude odds ratios with their corresponding 95% confidence limits. Two sample t-test was used to explore associations between continuous variables (age and body weight) and cure. A logistic regression model was built to analyse cure as a function of sex, age, initial body weight, previous treatment history, and the region where the patient was treated.

## Results

A total of 175 confirmed pulmonary MDR-TB patients were enrolled in the national MDR-TB treatment programme in the study period (Table 2). More than one half of patients were enrolled in the central region of Nepal surrounding the capital. Sixty-two percent of patients were male. Their median age was 30 years, and 84% of MDR-TB patients were aged between 20–50 years. No significant difference in age was noted between the sexes. At the time of enrolment, the patients' median weight was 46 kg (5 kg higher in males than females, p<0.05), with 129 (74%) patients weighing up to 50 kg, and therefore receiving the lower dosage of treatment. Sixteen patients (9%) weighed less than 36 kg at the time of treatment start. Most patients were failures of Category 2 treatment (87%). All, but one, patients were smear-positive.

**Table 2:** Characteristics of the study population.

Variable	Male (N = 108)	Female (N = 67)	All sexes (N = 175)
**Age (y)**			
Mean (SD)	35.4 (12.9)	30.8 (11.4)	33.6 (12.5)
Median (IQR)	31.5 (20.5)	28 (12)	30 (18)
**Body weight (kg)**			
Mean (SD)	48.6 (9.1)	43.6 (8.5)	46.7 (9.2)
Median (IQR)	47.5 (12)	42 (12)	46 (11)

Region	N	%	N	%	N	%
Eastern	12	11%	9	13%	21	12%
Central	68	63%	43	64%	111	63%
Western	12	11%	8	12%	20	11%
Mid-western	11	10%	4	6%	15	9%
Far-western	5	5%	3	4%	8	5%
**Previous history**						
Category 2 failure	97	90%	56	84%	153	87%
Category 1 failure	6	6%	4	6%	10	6%
New case	1	1%	2	3%	3	2%
Not specified	4	4%	5	7%	9	5%

SD = Standard Deviation; IQR = Inter Quartile Range.

A full description of the resistance patterns to first-line anti-TB drugs at baseline DST is given in Table 3. Of the 175 MDR-TB patients enrolled 5 (2.9%) were infected with strains resistant to 2 first-line drugs, 42 (24.0%) were infected with strains resistant to 3 first-line drugs, and 128 (73.1%) were infected with strains resistant to all 4 first-line drugs tested.

**Table 3:** Resistance to first-line anti-TB drugs at baseline DST.

Drugs	No. of patients	% of patients
Isoniazid, Rifampicin	5	2.9
Isoniazid, Rifampicin, Streptomycin	31	17.7
Isoniazid, Rifampicin, Ethambutol	11	6.3
Isoniazid, Rifampicin, Streptomycin., Ethambutol	128	73.1
Total MDR	175	100

**Figure 2:**
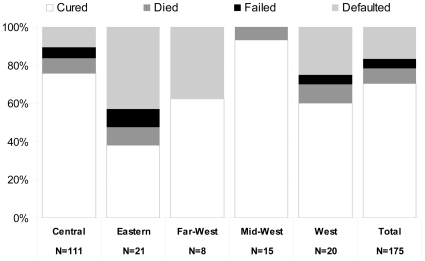
Treatment outcomes for MDR-TB cases by region, Nepal, 2005–2006.

**Figure 3:**
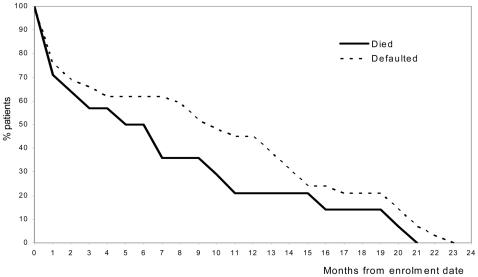
Months till death (N = 14, thick line) or default (N = 29, dotted line) for MDR-TB cases, Nepal, 2005–2006.

Most patients have been cured (123, 70%), 14 patients died (8%), 29 patients defaulted (17%), and nine patients failed (5%) (Figure 2). No statistically significant difference in the number of resistant drugs at baseline DST was found between patients who became cured and those who experienced an unfavorable treatment outcome (treatment failure, death, or default). The proportion cured varied substantially by region. Default accounted for much of the regional variation, with low default in the Central region (76% cure) and none in the mid-West (93% cure). The Eastern region had the lowest cure (38%) as a result of the highest proportion of patients defaulting and dying. At bivariate analysis, default was found to be associated with two regions with highest default [Eastern OR = 6.2; 95%CL2.0-18.9; Far West OR = 5.0; 95%CL1.0-24.3]. Cases who died had a lower mean body weight than those surviving (40.3 kg vs 47.2 kg, p<0.05; data not shown). One third of deaths and defaults happened within 2 months of enrolment, following which the occurrence of both outcomes was more or less constant throughout the remaining period of treatment (Figure 3).

The median duration of bacteriological monitoring and of treatment of cured cases was 730 days (range: 554–815 days, data not shown). Among the 123 who were cured, 112 (91%) experienced a durable sputum-smear conversion to negative within the first six months after treatment start. Patients who failed to be cured through the programme were treated for a median duration of 721 days (range: 404–959 days). Of the 14 patients who died, one converted to negative at month three and one at month seven after treatment while 12 patients were culture positive at the time of death. Adverse events recorded during treatment of this cohort of patients are presented in Table 4.

**Table 4:** Major adverse events registered in the study cohort.

Adverse events	No. of Patients	%
Nausea/Vomiting	80	64.0
Diarrhoea	1	0.8
Arthralgia	90	72.0
Dizziness/vertigo	12	9.6
Hearing disturbance	12	9.6
Visual disturbances	6	4.8
Hypothyroidism	1	0.8
Sleep disturbances and minor mood changes	12	9.6
Depression	7	5.6
Suicidal thoughts	7	5.6
Psychosis	4	3.2
Rash	17	13.6
Jaundice	5	4.0
Seizures	0	0.0
Gynaecomastia	4	3.2

Bivariate analysis showed a significant positive linear relationship between cure and initial body weight (p = 0.001). In the Eastern region, cure was significantly lower than in the Central (OR = 5.1, 95%CL1.8 - 14.2) and Mid-western (OR = 22.8, 95%CL1.5 - 340.0) regions. The same associations with body weight and with region became stronger after adjustment in the multivariable model (Table 5). No significant associations were otherwise detected with sex, age-group and between the four different treatment history categories.

**Table 5:** Variables associated with cure.

	Bivariate analysis		Multivariable analysis	
	Crude OR	95% CL	Adjusted OR	95% CL
**Sex**				
Male	1.0	Ref.	1.0	Ref
Female	1.0	0.5–2.1	1.6	0.7–3.7
**Age-group (y)**				
14–24	1.6	0.6–4.2	2.1	0.7–6.6
25–34	1.2	0.5–2.7	1.6	0.6–4.3
35–44	1.0	0.3–3.0	1.1	0.3–3.8
45–69	1.0	Ref.	1.0	Ref.
**Body weight (kg)**				
21–35	1.0	Ref.	1.0	Ref.
36–44	4.4	1.2–15.6	5.2	1.4–19.5
45–54	7.3	2.0–26.8	10.8	2.8–41.4
55–75	9.1	1.9–42.6	15.9	3.5–72.7
**Region**				
Eastern	1.0	Ref.	1.0	Ref.
Central	5.1	1.8–14.2	7.2	2.4–21.3
Far-western	2.7	0.5–15.6	4.6	0.7–28.1
Mid-western	22.8	1.5–340.0	31.2	3.2–307.5
Western	2.4	0.7–9.0	2.4	0.6–9.4
**Previous history**				
Category 2 failure	1.0	Ref.	1.0	Ref.
Category 1 failure	1.0	0.2–3.9	0.3	0.1–1.1
New case	0.8	0.1–9.5		
Not specified	0.8	0.2–3.5		

## Discussion

In this manuscript we describe the performance of an MDR-TB treatment program in a resource-limited setting designed around two main elements: a) integration of MDR-TB management in an existing ambulatory-based TB care delivery system; b) utilization of a fully standardized treatment regimen, i.e. DST results are not used to modify treatment regimens. To our knowledge the large majority of studies describing ambulatory- or community-based approaches to MDR-TB treatment adopt individualized treatment regimens, i.e. patients are offered an empiric treatment regimen which is tailored to the resistance patterns of the infecting strain as soon as DST results became available. Vast experience from Peru shows that community-based treatment for MDR-TB and even XDR-TB is feasible and can yield high cure rates [13], [14], [15]. The findings of our study go on the same direction: integrating MDR-TB treatment in a fully ambulatory-based TB care delivery system in resource-limited settings is possible with very good treatment outcome results. Compared to Peru the experience from Nepal shows us something additional: a standardized treatment regimen for MDR-TB, when designed based on good drug resistance surveillance data, can be successfully delivered in a resource-limited setting through an ambulatory-based system without the need of tailoring it to individual DST results. The cure rates documented in our study are quite high (70%) and in line with those reported in hospital-based care delivery systems or using individualized treatment regimens based on DST results [5]. Previous experiences have shown the feasibility of using fully standardized treatment regimens for MDR-TB but, in most circumstances, patients were hospitalized at least during the initial phase of treatment. In Bangladesh, for example, cure rates of 69% among MDR-TB patients were achieved using a fully standardized treatment regimen, but patients were asked to stay in hospitals for the first three months of treatment [12]. A fully standardized treatment regimen for MDR-TB was also used in the Republic of Korea; in that circumstance the therapy was self administered and treatment outcomes were very poor [16].

In Peru the only cohort of MDR-TB patients treated with a fully standardized regimen on ambulatory basis gave discouraging results, with only 57% of the patients being cured [17]. This sub-optimal outcome was probably due to the weak composition of the treatment regimen, designed without taking into proper consideration the underling resistance patterns to first- and second-line drugs in the population. In Nepal a well conducted nationwide drug resistance survey [9], [10] provided population level data on patterns of drug resistance that have been used to design a strong standardized regimen for MDR-TB which could be entirely delivered in outpatient facilities.

An individualized treatment regimen for MDR-TB would likely not have worked in our setting. The lack of experienced health workers on MDR-TB management and the long distances between TB treatment centers would not have made possible the expansion of MDR-TB services had it not been for the utilization of a fully standardized regimen. The utilization of a standardized regimen makes training, drug forecasting, and management of adverse events much easier, in addition to reducing significantly the overall cost of treatment [18].

The large majority of patients enrolled in Nepal were Cat 2 failures (87%), and there was no recruiting among previously untreated TB patients (except family contacts). The strict application of the enrolment criteria has made possible to use a standardized treatment regimen throughout the country.

Despite decentralization of the services in ambulatories in several regions overall default rates are quite high, but in line with previous publications from countries implementing similar programs [19]. Unfavorable outcomes are not evenly distributed across different areas of the country (Figure 2). The sub-optimal outcomes in the Eastern region is a result of poor performance at the NATA clinic in the region, which has a greater proportion of defaulters. The large majority of them interrupt treatment within the first six months from treatment start and after sputum and culture conversion (data not shown) when they start feeling better. The NATA clinic serves a very large catchment area and more MDR-TB clinics have been recently opened in that area to improve access to services in the most remote areas. In addition, to encourage adherence to treatment, monetary incentives for patients showing to take treatment regularly have been made available by the programme. It will be interesting to evaluate the effect of these incentives on default rates in the near future.

As expected, patients with low body weight have significantly higher rates of death than those weighing more. In this cohort the higher the body weight the greater was the chance of being cured (Table 5). These findings are in line with previous publications showing that undernourished patients with MDR-TB have greater risk of unfavorable treatment outcomes [20] and that malnutrition is an important risk factor for the development of TB [21]. In this cohort the correlation between body weight and cure rate is very strong. Food incentives and extra support should definitely be considered for MDR-TB patients weighting less than 36 Kg.

Adverse events were managed by local health workers using a standardized protocol based on the WHO guidelines [11], [22]. Monitoring of potassium and creatinine was carried out on monthly basis during the initial phase on treatment. In cases of low potassium, magnesium was also checked. Renal and hepatic functions were monitored only on the basis of symptoms. Type and frequencies of adverse events reported in this study don't differ from what previously reported on similar cohorts of patients [23].

A total of 8 patients had disease caused by NTM strains (Table 1) and ended up being treated with the standardized treatment regimen for MDR-TB used in the country given the absence of an alternative therapy. As expected the overall treatment outcomes have been poor. Only the 2 patients infected with *M.fortuitum* were cured. The remaining 6 patients (5 infected with *M.intracellulare* and 1 with *M.avium*) defaulted. The proportion of patients with NTM that end up being treated with regimens for MDR-TB might not be negligible in resource-limited settings. International guidelines on how to treat these patients are urgently needed. For the moment guidance documents have been produced by some medical societies [24].

One limitation of this study is that, due to lack of access to routine DST for second-line drugs, XDR-TB cases could not be detected at the start of MDR-TB treatment. Undiagnosed XDR-TB may therefore be the cause of death of treatment failure in some of the patients in this cohort. XDR-TB is though to be present in around 7% of the patients enrolled in the MDR-TB treatment programme in Nepal. Recently DST for key second-line drugs has become more available and there are plans in Nepal to make it available to all patients with confirmed MDR-TB.

The experience from the first year of the MDR-TB treatment programme in Nepal shows that fully standardized treatment for MDR-TB delivered through a network of outpatient facilities is not only feasible but can also yield high cure rates in resource-limited settings.
